# Differences in STEM doctoral publication by ethnicity, gender and academic field at a large public research university

**DOI:** 10.1371/journal.pone.0174296

**Published:** 2017-04-05

**Authors:** Rodolfo Mendoza-Denton, Colette Patt, Aaron Fisher, Andrew Eppig, Ira Young, Andrew Smith, Mark A. Richards

**Affiliations:** 1 Department of Psychology, University of California, Berkeley, Berkeley, California, United States of America; 2 Division of Mathematical and Physical Sciences, University of California, Berkeley, Berkeley, California, United States of America; 3 Office of Planning and Analysis, University of California, Berkeley, Berkeley, California, United States of America; 4 Office of Equity and Inclusion, University of California, Berkeley, Berkeley, California, United States of America; 5 Division of Social Sciences, University of California, Berkeley, Berkeley, California, United States of America; 6 Graduate Division, University of California, Berkeley, Berkeley, California, United States of America; 7 Department of Earth and Planetary Science, University of California, Berkeley, Berkeley, California, United States of America; International Nutrition Inc, UNITED STATES

## Abstract

Two independent surveys of PhD students in STEM fields at the University of California, Berkeley, indicate that underrepresented minorities (URMs) publish at significantly lower rates than non-URM males, placing the former at a significant disadvantage as they compete for postdoctoral and faculty positions. Differences as a function of gender reveal a similar, though less consistent, pattern. A conspicuous exception is Berkeley’s College of Chemistry, where publication rates are tightly clustered as a function of ethnicity and gender, and where PhD students experience a highly structured program that includes early and systematic involvement in research, as well as clear expectations for publishing. Social science research supports the hypothesis that this more structured environment hastens the successful induction of diverse groups into the high-performance STEM academic track.

## Introduction

Increasing the diversity of the professoriate in STEM fields is a national priority [1]. Doctoral education is the principal gateway to the professoriate, motivating research on how graduate education contributes to (or inhibits) STEM faculty diversity [2,3]. Entry into the professoriate involves a highly competitive selection process, wherein only a small percentage of candidates are ultimately offered academic positions at universities.

In this process, a candidate’s publication record is key. Although academic institutions differ in the specific weight they give to the various spheres of professional accomplishment, the publication record serves as the gold standard against which academic potential within the professoriate is judged. Publication is the currency that determines not only offers of employment, but also tenure and promotion decisions. This is particularly true at top-tier research universities, which produce a disproportionate number of future faculty, and hence disproportionately influence whether the STEM faculty workforce will become more diverse nationwide [4]. To more fully understand disparities in hiring at the level of the professoriate, it is thus important to assess whether disparities exist in the rates of publication in peer-reviewed outlets among graduate students in STEM fields. The question of disparities in publication at the graduate level is especially important given recent evidence that across STEM fields, there has been a marked shift towards first publications occurring in graduate school as opposed to post-Ph.D. [5]

Current scholarship on diversity at the graduate level has focused on the attitudes and biases of science faculty, which can affect student and job candidate evaluations [6,7], and on the unique financial, mentoring, and advocacy needs of students from underrepresented groups given systematic barriers to their success [8,9]. However, surprisingly little attention appears to have been devoted to the question of disparate doctoral publication rates, which might shed light on important disparities in the competitiveness of newly minted PhDs for the academic job market.

Here we address this key point of leverage by reporting on research that compares publication rates according to ethnicity, gender, and department within STEM doctoral programs at UC Berkeley. As a large public university that has granted more STEM PhDs over the past 10 years than any other US university [10], UC Berkeley is an ideal setting to compare differences in publication opportunities among students as a function of underrepresented minority (URM) status and gender. We further note that 8 of the 10 largest STEM PhD producers in the US are also large public universities. To this end, we draw from two extensive datasets on STEM graduate students at UC Berkeley, described below in two studies. Participants provided written consent to participate in Study 1; the project was reviewed and approved by UC Berkeley's Committee for the Protection of Human Subjects (CPHS) under protocol 2012-05-4347. Study 2 data was provided by UC Berkeley’s Graduate Division according to CPHS guidelines and thus no individual consent was sought; the project was reviewed and approved by UC Berkeley's CPHS under protocol 2016-10-9231.

## Study 1

### Materials and methods

The first dataset, the Berkeley Life in Science Survey (BLISS), was conducted in 2013–2014 and examines potential differences in publishing activity among students as a function of URM status and gender. BLISS was conducted as a baseline study prior to implementation of interventions intended to increase the success of diverse students in the mathematical, physical and computer sciences.

The Berkeley Life in the Sciences Study (BLISS) formed an initial step in establishing the Berkeley Science Network, funded by the Kapor Center for Social Impact (a nonprofit organization), and the Berkeley Science Connections Program, funded by the US National Science Foundation. These programs are designed to strengthen the pipeline of underrepresented students in the mathematical, physical, and computer sciences (MPCS) by strengthening connections among undergraduate, graduate, post-doctoral, and faculty scholars in these disciplines. We surveyed graduate students in the Division of Mathematical and Physical Sciences (mathematics, statistics, physics, astronomy, earth and planetary science), the department of electrical engineering and computer science (EECS), and the College of Chemistry (chemistry and chemical engineering). As a result, BLISS included MPCS fields and did not include other STEM disciplines such as biological sciences and other engineering fields. BLISS sampled from the population of students at UC Berkeley enrolled in doctoral programs in the sciences, randomly sampling within ethnic and gender groups, while oversampling based on minority ethnic status and female gender.

Students eligible for inclusion as participants were identified by the university registrar. Survey completion was voluntary and garnered high participation rates. Table 1 lists participation rates for the BLISS survey. The use of unique participant identifiers enabled the researchers to link automatically to the students' records for demographic and enrollment and educational progress data (e.g., gender, ethnicity, GPA, educational status, years of graduate student teaching and research employment).

**Table 1:** Participation rates for the Berkeley Life in Science Survey (BLISS).

	*Total*	*Completers*	*Percent*
Non-URM men	555	218	39%
Women	383	204	53%
URM	109	55	50%
M&PS	398	165	41%
EECS	234	88	38%
Chemistry	381	199	52%

The survey itself contained a battery of questions designed to address various aspects of graduate student life in the sciences at Berkeley. Although the survey was not originally designed to address publication disparities per se, we did include a question in which the students indicated whether they had submitted a manuscript for publication in the past year. Focusing on manuscript submission rather than submission outcome (i.e., rejection, or acceptance) addresses possible disparities in student engagement in the process of publishing independently of the external peer review process (i.e., whether the paper was accepted or rejected). The question has binary response options (*yes*/*no*), and was thus treated as a Bernoulli process. We benchmark the comparisons of URM and female graduate students against male non-URM students, which includes White as well as Asian background males and for whom no statistically significant differences emerged in the two datasets reported here.

## Results

Table 2 provides headcounts, observed responses, percent of affirmative responses, standard errors, confidence intervals, and p-values (compared to non-URM men) for self-reported submission of a paper for publication in the BLISS survey. The aggregate of these responses for each question is described with binomial statistics. The probability of success, p, is the percentage of yes responses to a given question. Standard errors are calculated with pooled binomial standard errors [11]. Confidence intervals are calculated using the Clopper-Pearson interval [12]. P-values are calculated using a two-tailed exact binomial test [13].

**Table 2:** Self-reported submission of a paper for publication (Study 1).

Division	Student group	n	Observed		% Yes	SE	95% CI		p-value
			No	Yes			Lower	Upper	
All	Non-URM men	181	105	76	42%	4%	35%	50%	
	Women	192	130	62	32%	5%	26%	39%	0.20
	URM	52	40	12	23%	8%	13%	37%	0.03
EECS+MPS	Non-URM men	115	57	58	50%	5%	41%	60%	
	Women	83	56	27	33%	7%	23%	44%	0.06
	URM	29	24	5	17%	12%	6%	36%	0.00
Chemistry	Non-URM men	66	48	18	27%	5%	17%	40%	
	Women	109	74	35	32%	7%	23%	42%	0.68
	URM	23	16	7	30%	11%	13%	53%	0.82

**Fig 1:**
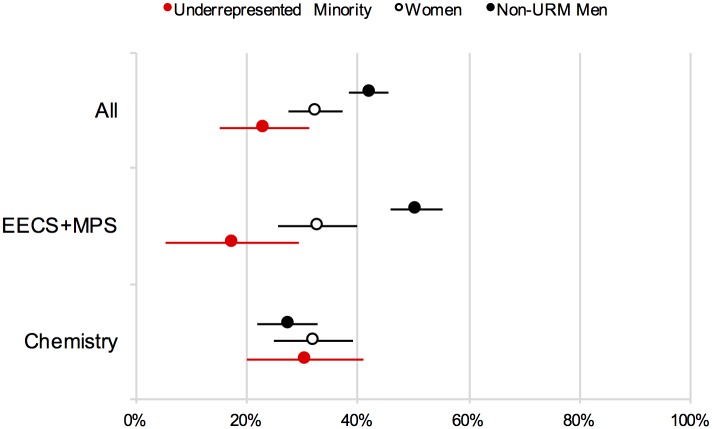
Self-reported submission of a paper for publication (Study 1). Note: Error bars represent ±1 SE.

Fig 1 illustrates the findings graphically. As seen in the top row of Fig 1, across the entire sample, URM students were only about half as likely to have submitted a paper in the last year. The gender disparity is about half of this. We then asked whether this disparity was evident across the departments surveyed. As seen in the middle panel of the figure, in the combined sample of MPS and EECS departments (aggregated to protect participant privacy given small sub-group samples), both female and URM students reported lower rates of having submitted a publication in the past year, compared to male, non-URM students (this is also true for MPS and EECS individually). As the bottom panel of Fig 1 illustrates, however, in the College of Chemistry there were no significant differences between female or URM students and their male, non-URM counterparts. These results were completely unanticipated at the outset of the survey.

We additionally used logistic regression to statistically capture whether the effect of URM status or gender differed in Chemistry versus other departments. Logistic regression is used in cases where the dependent variable (in this case, submission of a paper for publication) is a dichotomous variable; multiple logistic regression is used to understand the unique effect of a given set of measured or independent variables in predicting the dependent variable [14].

Table 3 shows the results from a multiple logistic regression estimating the likelihood (in log odds) that a graduate student submitted a manuscript for publication as a function of gender (0 = male, 1 = female), underrepresented minority (URM) status (0 = non-URM, 1 = URM), Chemistry affiliation (0 = not in Chemistry, 1 = in Chemistry), and the two-way interactions for Chemistry with gender and URM status. As the table shows, the results revealed significant effects of URM status (URM), gender (Gender), and Chemistry affiliation (Chem). These main effects are qualified by a significant interaction between URM status and chemistry, reflecting the fact that the negative effect of URM status on publication is offset by Chemistry affiliation. A similar, albeit non-significant, interaction pattern is observed for Chemistry affiliation and gender.

**Table 3:** Logistic regression model for submitting a paper for publication.

	Estimate	Std. Error	z value	p value
(Intercept)	0.051	0.185	0.276	0.782
URM (0,1)	-1.647	0.564	-2.922	0.003
Gender (0,1)	-0.641	0.298	-2.148	0.032
Chem (0,1)	-0.926	0.319	-2.905	0.004
URM:Chem	1.615	0.743	2.173	0.030
Gender:Chem	0.772	0.442	1.746	0.081

We sought to ensure that the observed findings were not due to underlying differences among the student populations being compared. In a second multiple logistic regression model, we therefore added to the above model four variables likely to affect a student’s publication efforts. First, we controlled for the number of years the student had been in the program (one student who reported having been in the graduate program for 38 years was excluded from this analysis, though his/her inclusion does not affect the findings). We also controlled for time spent employed in research, teaching, and on fellowship. These last three variables were converted to a fraction of the time spent in the program. Table 4 provides the results of this analysis. The results show that being in Chemistry (Chem), URM status (URM), and their interaction remain robust predictors of whether a student submitted a paper for publication, even when controlling for years enrolled in graduate school (Time), research employment (RA), teaching assistantships (TA), and fellowship support (Fellowship). However, the effect of gender becomes attenuated in this analysis.

**Table 4:** Logistic regression model for submitting a paper for publication, with covariates.

	Estimate	Std. Error	z value	p value
(Intercept)	-1.625	0.622	-2.612	0.009
Time	0.550	0.107	5.143	<0.001
RA	0.479	0.287	1.668	0.095
TA	-0.703	0.313	-2.248	0.025
Fellowship	0.788	0.259	3.041	0.002
URM (0,1)	-2.189	0.657	-3.334	0.001
Chem (0,1)	-0.730	0.361	-2.025	0.043
Gender (0,1)	-0.892	0.341	-2.615	0.009
URM:Chem	1.711	0.853	2.005	0.045
Gender:Chem	0.860	0.492	1.750	0.080

The possibility remains that the students who volunteered to participate in this survey were not representative of all PhD students at the University. Further, data collection was limited to a single year, raising a concern that the disparities observed may not reflect long-term trends. To address these limitations, we examined a second dataset at UC Berkeley, the Graduate Division Exit Survey, required of all students prior to being granted their PhDs. The survey has been administered since 1995 with a response rate of 98%. The survey is retrospective and covers aspects of doctoral student experience over the whole period of students’ degree programs at Berkeley.

## Study 2

### Materials and methods

The PhD Exit Survey is administered at the time of degree completion. Doctoral candidates submit the survey at the time that they file their paperwork with the Graduate Division. Candidates are not required to submit the survey form, but it is on the checklist of paperwork to be completed at the time of filing. Items on the survey are grouped into sections covering financial support, quality of advising, relationship with dissertation chair, aspects of scholarly practice, and first placement. The data for this study were extracted from the database, limiting responses to students who identified their majors in the broad disciplinary areas of biological, physical, and social sciences, and engineering in the academic years spanning 1998–1999 to 2013–2014. The overall completion rate for the time period of this study was 98%. UC Berkeley’s Graduate Division does not receive external funding for this survey.

We include data from the most recent 15-year period (1998–2013), allowing us to examine potential publication disparities with larger sample sizes, and thus examine trends separately for EECS, Mathematics, and Physics, three of the largest departments in the university. Note that this survey includes the Biological Sciences. Table 5 provides summary statistics for completers of this survey.

**Table 5:** PhD survey participants (PhD exit survey).

Division	Total	Non-URM Men	Women	URM
Bio	1,563	690	812	103
Chemistry	1,273	814	415	66
EECS	692	559	107	22
MPS	1,242	939	244	59
Mathematics	377	298	55	26
Physics	502	408	65	23
All	4,770	3,002	1,578	250

Note: As categories are not mutually exclusive (i.e., URM women are included in both the Women and URM cells), row totals do not necessarily equal row n’s. EECS = Electrical Engineering and Computer Science. MPS = Mathematics and Physical Sciences.

The PhD exit survey contained two questions that are particularly relevant to our analysis. The first asked students, “Did you deliver any papers at national scholarly meetings?” Presenting at national meetings is an important precursor to publication and signals active engagement in the research enterprise [15]. A second question in this survey asked, "Were you encouraged by faculty in your department to publish?" As with BLISS, the responses have binary (*yes/no*) response options and were thus analyzed similarly. We discuss findings and analyses for each of these questions in turn.

## Results

**Fig 2:**
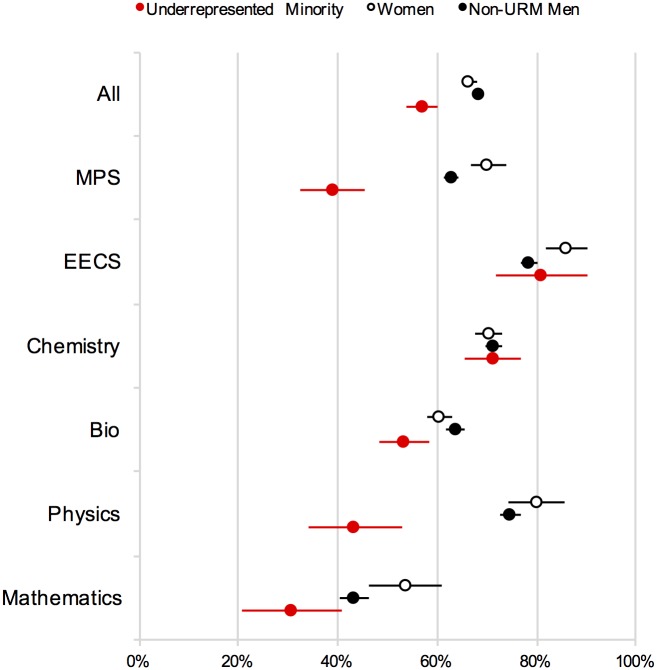
Delivery of papers at national scholarly meetings (Study 2). Note: Error bars represent ±1 SE.

Table 6 presents headcounts, observed responses, percent of affirmative responses, standard errors, confidence intervals, and p-values (compared to non-URM men) for the question, “Did you deliver any papers at national scholarly meetings?” Fig 2 presents illustrates the data from Table 6 graphically.

**Table 6:** Delivery of papers at national scholarly meetings (Study 2).

Division	Student group	n	Observed		% Yes	SE	95% CI		p-value
			No	Yes			Lower	Upper	
All	Non-URM Men	2985	948	2037	68%	1%	67%	70%	
	Women	1572	528	1044	66%	1%	64%	69%	0.12
	URM	249	107	142	57%	3%	51%	63%	0.00
MPS	Non-URM Men	936	348	588	63%	2%	60%	66%	
	Women	242	72	170	70%	3%	64%	76%	0.02
	URM	59	36	23	39%	7%	27%	53%	0.00
EECS	Non-URM Men	552	119	433	78%	2%	75%	82%	
	Women	107	15	92	86%	4%	78%	92%	0.06
	URM	21	4	17	81%	9%	58%	95%	1.00
Chemistry	Non-URM Men	811	232	579	71%	2%	68%	74%	
	Women	415	123	292	70%	3%	66%	75%	0.66
	URM	66	19	47	71%	6%	59%	82%	1.00
Biology	Non-URM Men	686	249	437	64%	2%	60%	67%	
	Women	808	318	490	61%	3%	57%	64%	0.07
	URM	103	48	55	53%	5%	43%	63%	0.03
Physics	Non-URM Men	407	103	304	75%	2%	70%	79%	
	Women	65	13	52	80%	6%	68%	89%	0.39
	URM	23	13	10	43%	10%	23%	66%	0.00
Math	Non-URM Men	297	168	129	43%	3%	38%	49%	
	Women	54	25	29	54%	7%	40%	67%	0.13
	URM	26	18	8	31%	10%	14%	52%	0.24

Table 7 shows the results from a multiple logistic regression modeling the likelihood (in log odds) of a graduate student delivering a paper at a national conference as a function of gender (Gender), underrepresented minority status (URM), Chemistry affiliation (Chem), and the two-way interactions for Chemistry with gender and URM status. As the table shows, the results revealed significant effects of URM status and Chemistry affiliation. These main effects are qualified by a significant interaction between URM status and chemistry, reflecting the fact that the negative effect of URM status on publication is offset by Chemistry affiliation. No main effects or interactions with gender were observed.

**Table 7:** Logistic regression model for delivery of papers at national scholarly meetings.

	Estimate	Std. Error	z value	p value
(Intercept)	0.713	0.045	15.912	<.001
Chem (0,1)	0.198	0.089	2.234	0.026
URM (0,1)	-0.616	0.153	-4.034	<0.001
Gender (0,1)	-0.054	0.076	-0.705	0.481
URM:Chem	0.630	0.318	1.979	0.048
Gender:Chem	0.006	0.152	0.037	0.971

Following our analytic strategy, we ran an additional multiple logistic regression that added time spent in the program (Time), research employment (RA), teaching assistantships (TA), and fellowships (Fellowship) as covariates, with the last three expressed as a fraction of time spent in the program. Table 8 provides details of this analysis, which shows that the critical URM status by Chemistry affiliation interaction remains robust in the presence of these covariates.

**Table 8:** Logistic regression model for delivery of papers at national scholarly meetings, with covariates.

	Estimate	Std. Error	z value	p value
(Intercept)	1.148	0.163	7.064	<0.001
Time	-0.052	0.020	-2.579	0.010
RA	0.113	0.041	2.785	0.005
TA	-0.336	0.055	-6.091	<0.001
Fellowship	0.180	0.073	2.479	0.013
Chem (0,1)	0.214	0.091	2.356	0.018
URM (0,1)	-0.644	0.156	-4.127	<0.001
Gender (0,1)	-0.095	0.077	-1.231	0.218
URM:Chem	0.644	0.320	2.013	0.044
Gender:Chem	0.046	0.153	0.299	0.765

**Fig 3:**
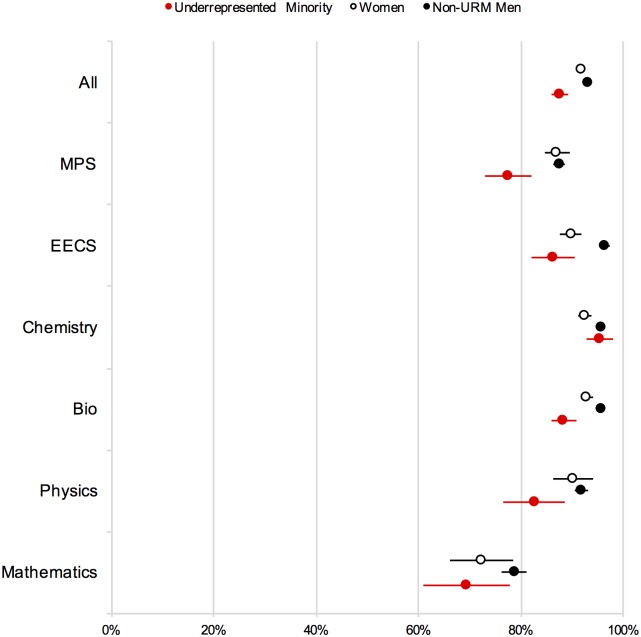
Encouragement by faculty to publish (Ph.D. exit survey). Note: Error bars represent ±1 SE.

Table 9 presents headcounts, observed responses, percent of affirmative responses, standard errors, confidence intervals, and p-values (compared to non-URM men) for the item, "Were you encouraged by faculty in your department to publish?" The data correspond to Fig 3.

**Table 9:** Encouragement by faculty to publish (Study 2).

Division	Student group	n	Observed		% Yes	SE	95% CI		p-value
			No	Yes			Lower	Upper	
All	Non-URM Men	2983	203	2780	93%	0%	92%	94%	
	Women	1561	130	1431	92%	1%	90%	93%	0.02
	URM	249	31	218	88%	2%	83%	91%	0.00
MPS	Non-URM Men	932	117	815	87%	1%	85%	90%	
	Women	238	31	207	87%	2%	82%	91%	0.84
	URM	58	13	45	78%	5%	65%	87%	0.04
EECS	Non-URM Men	554	20	534	96%	1%	94%	98%	
	Women	107	11	96	90%	2%	82%	95%	0.00
	URM	22	3	19	86%	4%	65%	97%	0.04
Chemistry	Non-URM Men	811	36	775	96%	1%	94%	97%	
	Women	411	31	380	92%	1%	89%	95%	0.01
	URM	66	3	63	95%	3%	87%	99%	0.77
Biology	Non-URM Men	686	30	656	96%	1%	94%	97%	
	Women	805	57	748	93%	1%	91%	95%	0.00
	URM	103	12	91	88%	2%	81%	94%	0.00
Physics	Non-URM Men	403	33	370	92%	1%	89%	94%	
	Women	61	6	55	90%	4%	80%	96%	0.64
	URM	23	4	19	83%	6%	61%	95%	0.11
Math	Non-URM Men	296	63	233	79%	2%	74%	83%	
	Women	54	15	39	72%	6%	58%	84%	0.25
	URM	26	8	18	69%	8%	48%	86%	0.23

Table 10 shows the results from a multiple logistic regression modeling the likelihood (in log odds) of a graduate student being encouraged to publish as a function of gender (Gender), URM status (URM), Chemistry affiliation (Chem), and the two-way interactions for Chemistry with gender and URM status. As the table shows, the results revealed significant main effects of URM status (negative) and Chemistry affiliation (positive). The interaction terms involving Chemistry affiliation and identity (URM status and gender) were not significant in this model, reflecting reduced variability in encouragement to publish relative to presentation at national meetings (see Fig 3). Nonetheless, we note a pattern of tighter clustering in Chemistry in comparison to other departments.

**Table 10:** Logistic regression model for encouragement by faculty to publish.

	Estimate	Std. Error	z value	p value
(Intercept)	2.457	0.078	31.624	<.0001
Chem (0,1)	0.573	0.183	3.136	0.002
URM (0,1)	-0.732	0.215	-3.401	0.001
Gender (0,1)	-0.035	0.130	-0.271	0.786
URM:Chem	1.009	0.643	1.570	0.116
Gender:Chem	-0.505	0.281	-1.801	0.072

Finally, we also ran a multiple logistic regression predicting encouragement to publish that included all of the above variables but also added time spent in the program (Time), research employment (RA), teaching assistantships (TA), and fellowships (Fellowship) as covariates, with the last three expressed as a fraction of time spent in the program. Table 11 provides details of this analysis, which shows the main effects of interest and interactions unchanged.

**Table 11:** Logistic regression model for encouragement by faculty to publish, with covariates.

	Estimate	Std. Error	z value	p value
(Intercept)	4.034	0.289	13.971	<0.001
Time	-0.205	0.032	-6.433	<0.001
RA	0.097	0.074	1.306	0.192
TA	-0.567	0.093	-6.099	<0.001
Fellowship	0.271	0.143	1.900	0.058
Chem (0,1)	0.503	0.186	2.704	0.007
URM (0,1)	-0.741	0.226	-3.275	0.001
Gender (0,1)	-0.146	0.133	-1.100	0.271
URM:Chem	1.000	0.647	1.545	0.122
URM:Gender	-0.417	0.282	-1.476	0.140

The exit survey data replicate the finding that URM students in particular are under-encouraged to publish and are provided fewer opportunities to present their research than their non-URM male counterparts. We also observe a more modest and less consistent gender disparity, as in the BLISS survey. The data also reveal that the College of Chemistry is more successful than the other departments in the STEM fields at Berkeley in mitigating disparities in presentation opportunities between URM and majority male PhD students.

## Discussion

Does Chemistry have a different approach to graduate education, or specifically to helping students work toward publication of their research, than other disciplines at the university? We have begun to explore this question by considering the formal requirements and conventional practices of the graduate programs in the departments included in this study.

In Chemistry, we find that students experience a highly-structured environment in which they are introduced to research (via lab rotations) at the outset of their studies, their advisors are regularly and systematically queried as to their students’ progress, and expectations surrounding publication of research results are both implicitly and explicitly clear even in the first two years of study. We also note that Berkeley’s chemistry department has been particularly successful in placing their women PhDs in prestigious academic positions, relative to their peers [16]. By contrast, in Berkeley’s departments of mathematics and physics, students report a relatively weakly structured environment compared with that in chemistry. However, further research is needed to better understand how these factors play out among other STEM departments.

Research in education and psychology suggests that a lack of structure and/or clear expectations will have a disproportionate effect upon students who come to graduate school less familiar with a high-performance, research-oriented academic environment—e.g., first generation college graduates, whose parents are neither professionals nor academics, and students from non-research-oriented colleges [17]. For these students, unstated assumptions regarding the norms for academic productivity (publishing, presentations at prestigious conferences, etc.) may not become apparent to them until late in their PhD studies. Also, to the degree that the process of publishing often calls for subjective evaluation of the quality of a student's work, subtle judgments on the part of advisors and co-authors may cumulatively lead to fewer opportunities for minority scholars to present nationally and publish their work [18]. Virtually all of the steps involved in publishing an academic paper provide opportunities for the expression of subtle or unconscious bias- from the evaluation of an idea, to the procedures required to test those ideas, to deciding when a set of results is ready for publication, to the manuscript writing itself.

Research on diversifying Chemistry [17] and STEM more generally [19,20] has focused largely on recruitment of women and URM students into graduate programs, as well as these students’ progress towards (and completion of) the Ph.D. This research highlights several important best practices for recruitment and retention efforts, including a visible commitment from institutional administrators, targeted scholarships, strong mentoring, and systematic benchmarking of both student progress and institutional goals. It remains an open question whether the best practices for recruitment and retention of underrepresented students in STEM fields lead to equity in research productivity. To fully understand disparities in hiring at the level of the professoriate, it is necessary to move beyond comparisons of normative student outcomes (e.g., graduation rates), and to assess instead whether disparities exist in the rates of publication in peer-reviewed outlets as graduate students in STEM fields consider and enter the academic job market. We underscore publications as a key factor that needs to be taken into account for increasing diversity within the professoriate.

Future research will need to address whether the findings observed at Berkeley are representative of Chemistry departments more generally, or whether they represent a specific culture that has been nurtured at Berkeley but which is nevertheless potentially replicable. Nonetheless, these findings suggest that straightforward measures to provide PhD students in STEM with well-structured environments, which should in fact be beneficial to all students [21], may mitigate against confounding issues of under-preparation and bias that might otherwise impede efforts to diversify the professoriate, especially at research-oriented universities.
